# Efficacy of infrared irradiation at predefined acupoints combined with task-oriented training as a rehabilitation strategy in cerebral infarction patients with hemiplegia

**DOI:** 10.3389/fneur.2026.1777129

**Published:** 2026-07-17

**Authors:** Yinyue Gu, Zhenwen Liang, Min Han, Jingwen Shao

**Affiliations:** 1 Department of Rehabilitation Medicine, Pudong Gongli Hospital, Shanghai University of Medicine & Health Sciences, Shanghai, China; 2 College of Rehabilitation Sciences, Shanghai University of Medicine & Health Sciences, Shanghai, China

**Keywords:** cerebral infarction, hemiplegia, infrared irradiation, limb function, task-oriented training

## Abstract

**Objective:**

Infrared irradiation could improve local blood circulation, regulate muscle metabolism and enhance neuromuscular excitability in stroke patients. This study aimed to investigate the effects of infrared irradiation at predefined acupoints (IPA) combined with task-oriented training (TOT) on neurological function, limb motor recovery, electromyographic activity, daily living ability, and quality of life in cerebral infarction patients with hemiplegia.

**Methods:**

A total of 208 patients with cerebral infarction with hemiplegia were included. Patients were randomly allocated to the IPA + TOT group (*n* = 105) or TOT-only group (*n* = 103) according to their rehabilitation method. Neurological impairment (NIHSS), limb motor function (FMA), electromyographic parameters (iEMG, RMS), activities of daily living (Barthel Index), and quality of life (SS-QOL) were evaluated at baseline and week 3 (W3).

**Results:**

At W3, the IPA + TOT group presented greater improvements in the Barthel index (*p* = 0.002), FMA-upper limb score (*p* = 0.025) and FMA-lower limb score (*p* = 0.003) than did the TOT-alone group. Electromyographic activity increased notably in the triceps brachii (iEMG: *p* = 0.035; RMS: *p* = 0.001) and tibialis anterior (iEMG: *p* = 0.037; RMS: *p* = 0.022) regions. The quality of life measured by the SS-QOL was also greater in the IPA + TOT group (*p* = 0.012). The NIHSS score was not significantly different between the groups.

**Conclusion:**

IPA combined with TOT is associated with improvements in motor function, muscle activation, daily living ability, and quality of life, supporting its potential as an effective complementary rehabilitation strategy for cerebral infarction patients with hemiplegia. However, further studies with longer follow-up periods and more rigorous study designs are required to confirm its long-term efficacy and clinical value.

## 1 Introduction

Cerebral infarction is one of the main causes of long-term disability in adults, among which hemiplegia is the most common manifestation of functional impairment (1, 2). Hemiplegic patients often suffer from decreased limb motor function, impaired neurological function and a reduced ability to perform daily activities, which impairs the rehabilitation process and reduces quality of life (3–5). Besides, patients with disability require intensive and long-term healthcare, rehabilitation and social support, which further increases the disease burden both in hemiplegic patients and their families (6). Task-oriented training (TOT) emphasizes functional and goal-oriented activities, which can promote motor reconstruction and is widely used in stroke rehabilitation (7, 8). TOT-based rehabilitation regimens, such as TOT combined with force feedback hand rehabilitation robots and repetitive transcranial magnetic stimulation, have been preliminarily explored and could further improve limb function in hemiplegia patients compared with TOT alone (9–11). However, more rehabilitation methods are needed to further increase neural activation and muscle participation in cerebral infarction patients with hemiplegia.

Infrared irradiation, as a safe and noninvasive physical therapy method, has gradually attracted attention in recent years. Previous studies have suggested that infrared irradiation can improve local blood circulation, regulate muscle metabolism and enhance neuromuscular excitability (12–15). Furthermore, recent study shows that photobiomodulation induced by infrared irradiation may promote neuronal energy metabolism and neuroplasticity through mitochondrial activation, which indicates an important role in the recovery of stroke (16). In addition, the predefined acupoints used in this study, including Jianyu (LI15), Quchi (LI11), Zusanli (ST36), Huantiao (GB30), Taichong (LR3), Sanyinjiao (SP6), and Yanglingquan (GB34), are commonly applied in post-stroke rehabilitation and have been associated with motor function recovery and neuroplasticity in previous studies (17, 18). Therefore, it was hypothesized that infrared irradiation at predefined acupoints (IPA) may provide additional benefits beyond conventional rehabilitation training.

Although current studies preliminary explores that several adjunctive rehabilitation strategies combined with TOT could improve the functional and neural outcomes (9–11) the clinical evidence regarding IPA combined with TOT for the rehabilitation of cerebral infarction patients with hemiplegia is still relatively limited. Therefore, this study adopted a randomized controlled design to explore the effects of IPA combined with TOT on neurological function, limb motor function, electromyography signals, activities of daily living and quality of life in cerebral infarction patients with hemiplegia, thereby providing a basis for the application of this combined protocol in clinical rehabilitation.

## 2 Methods

### 2.1 Subjects

A total of 208 cerebral infarction patients with hemiplegia who received rehabilitation treatment in the rehabilitation ward of our hospital from April 2024 to March 2025 were included in this study. The inclusion criteria were as follows: (1) the time since the first cerebral infarction occurred within 30 days and the vital signs of the patients were stable; (2) with unilateral hemiplegia; (3) Patients who were 18 to 80 years old; (4) patients with no obvious cognitive dysfunction; (5) patients who did not participate in other studies during the same period; (6) patients and their family members who voluntarily participated and signed the informed consent form. They lived in Shanghai and could cooperate with the follow-up. The exclusion criteria were as follows: (1) patients with epilepsy or severe failure of vital organs such as the heart, liver, kidneys, and hematopoietic system; (2) patients with skin damage or those who cannot tolerate infrared radiation; and (3) patients with congenital limb deformities or severe scoliosis. Those patients were excluded from the final analysis: (1) those with recurrent cerebral infarction or sudden other cardiovascular and cerebrovascular events during the intervention period; (2) those whose interventions were interrupted or who did not follow the doctor’s advice to receive other rehabilitation treatments; and (3) researchers who were lost to follow-up or withdrawn halfway. The Medical Ethics Committee of Pudong Gongli Hospital, Shanghai University of Medicine & Health Sciences approved this study, with approval number GLYY1s2024-042.

### 2.2 Randomization and blinding

The block randomization method was applied for grouping, with the block size set at 6. The grouping results were sealed and saved by the study coordinator, who was not related to the intervention implementation and was unblinded and assigned in sequence after the patients completed the baseline assessment. Owing to the nature of the intervention measures, the rehabilitation therapists implementing the treatment and the subjects were not blinded, but single-blind methods were applied to both the assessors and the data analysts to reduce subjective bias.

### 2.3 Intervention

The control group received TOT intervention. The training consisted of two parts: upper limb and lower limb functional training. (1) Upper limb function training: When the patient lay in a supine position, the rehabilitation therapist assisted the patient in touching the opposite shoulder, mouth, forehead and other areas using their affected upper limb or straightened their body and raised the upper limb to reach the target set by the therapist. When the patient was in a sitting position, patients were asked to use the affected upper limb to push building blocks, plastic blocks and other objects to target sites in different directions or to grasp objects and move them from the designated position to another place. When conducting reaching object training in the sitting position, under the stable sitting posture, patients were asked to raise their upper limbs in different directions to complete the prespecified task. During the training process, weight-bearing, movement distance, and joint range of motion gradually increased, with a focus on strengthening actions such as flexion, abduction, external rotation, elbow extension, wrist extension and finger extension, to achieve progressive functional improvement. (2) Lower limb function training: Patients were asked to perform task-oriented movements such as knee flexion, hip adduction, and abduction under the guidance of a therapist. The target points were set for each movement, and patients were required to focus their attention on the target tasks themselves rather than merely on limb movements. All interventions were completed under the “one-on-one” guidance of rehabilitation therapists. The intervention frequency was 30 to 50 min per session, once a day, 5 days a week, and the continuous intervention lasted for 3 weeks.

Patients in the experimental group were treated with IPA in combination with TOT. The IPA was administered as follows: an infrared multifrequency laser therapy device was used. The detail parameters were as follows: (1) wavelength (800 nm), output power (40 mW), irradiation distance (10–15 cm); (2) The predefined acupoints were selected before study initiation according to commonly used post-stroke rehabilitation protocols and included Jianyu (LI15), Quchi (LI11), Zusanli (ST36), Huantiao (GB30), Taichong (LR3), Sanyinjiao (SP6), and Yanglingquan (GB34); (3) treatment frequency: each acupoint was irradiated for 10 min or 5 times a week; (4) total treatment duration: intervention was carried out continuously for 3 weeks. During treatment, the patient should be in a comfortable position. The irradiation distance should be maintained at 10 to 15 cm. The local skin should be warm without a burning sensation. During the treatment period, all irradiation parameters were recorded by qualified rehabilitation therapists.

### 2.4 Evaluations

The National Institutes of Health Stroke Scale (NIHSS) score was used for the assessment of neurological impairment. This scale consists of 11 items, including consciousness, visual field, facial paralysis, upper limb movement, lower limb movement, ataxia movement, sensation, language, articulation and neglect, with a total score of 42 points. The higher the score is, the more severe the degree of neurological function impairment. The Fugl–Meyer assessment (FMA) is used to evaluate limb motor function and includes two parts, the upper limb (66 points) and the lower limb (34 points), with a total of 17 items and a total score of 100 points. The higher the score was, the better the motor function. The surface electromyographic signals of the triceps brachii, extensor carpi ulnaris, and tibialis anterior were detected via an electromyographic signal acquisition system. The triceps brachii, wrist extensor muscles, and tibialis anterior were selected because they represented proximal upper-limb, distal upper-limb, and lower-limb motor function, respectively. Besides, these muscles were commonly used for electromyographic assessment in stroke rehabilitation studies (19, 20). Surface electromyography was recorded using an SG-MINI-8 system (Zhejiang Dinuo Medical Technology Co., Ltd., China) with disposable surface electrodes (E5). The sampling frequency was set at 2000 Hz, and signals were processed using a band-pass filter of 5–500 Hz (Reason for this range: this filtering range was commonly used to attenuate motion artifacts and baseline drift at low frequencies while reducing high-frequency noise and preserving the relevant EMG signal components). In addition, a built-in 50 Hz notch filter of the SG-MINI-8 system was applied to suppress power-line interference during signal acquisition and processing. Electrodes were placed longitudinally over the muscle belly along the direction of the muscle fibers. Integrated electromyography (iEMG) and root mean square (RMS) values were calculated for quantitative analysis. The signal was wirelessly transmitted to the computer and processed and analyzed via the system’s built-in software. No MVC normalization was performed, and all measurements were conducted under the same standardized testing conditions before and after treatment. The modified Barthel index was used for the assessment of activities of daily living, covering 10 items, including eating, dressing, bathing, using the toilet, walking, controlling defecation and urination, going up and down stairs, bed and chair transfer and grooming, with a total score of 100. The higher the score is, the stronger the ability to take care of oneself in daily life. The stroke-specific quality of life (SS-QOL) scale was used to assess quality of life, including 12 dimensions, such as energy, family role, social role, activity, language, personality, self-care ability, upper limb function, vision, work/labor, thinking and emotion, with a total of 49 items. According to the Likert 5-point rating system (1 to 5 points), the total score ranges from 49 to 245 points. The higher the score was, the better the quality of life. The primary outcomes were Barthel Index and Fugl–Meyer Assessment scores. Secondary outcomes included NIHSS score, SS-QOL score, and electromyographic parameters.

### 2.5 Statistical analysis

R software (version 4.4.2; R Foundation for Statistical Computing, Vienna, Austria), SPSS version 26.0, and GraphPad Prism version 8.0.2 were used for statistical analysis and figure drawing. Continuous variables were first assessed for normality using the Kolmogorov–Smirnova test. Normally-distributed continuous variables data were presented as the means ± standard deviations, while non-normally distributed data are presented as median (interquartile range). Categorical variables are presented as numbers (percentages). Comparisons of data between groups were carried out via Student’s *t* test or Mann–Whitney *U* test as appropriate. Comparisons of categorical variables between groups were carried out via chi-square test. The comparison within groups from baseline to week 3 were performed by paired *t*-tests or Wilcoxon signed-rank test as appropriate. To control for the risk of type I error, the Benjamini–Hochberg false discovery rate (FDR) procedure was performed. Due to that these variables included in the correlation analyses did not satisfy the assumption of normality, Spearman’s correlation analysis was used to assess associations between continuous variables. Correlation coefficients and corresponding *p* values were calculated and visualized using correlation heatmaps. Repeated-measures analysis of variance (ANOVA) was performed to further evaluate whether the intervention effects differed among the evaluated muscles. When the assumption of sphericity was violated, Greenhouse–Geisser correction was applied. *p* < 0.05 was considered statistically significant.

## 3 Results

### 3.1 Patient characteristics

In total, 105 cerebral infarction patients with hemiplegia were included in the IPA + TOT group, while 103 patients were included in the TOT group. The mean ages were 64.6 ± 8.7 and 63.0 ± 9.3 years in the IPA + TOT and TOT groups, respectively. There were 70 (66.7%) males and 35 (33.3%) females in the IPA + TOT group and 66 (64.1%) males and 37 (35.9%) females in the TOT group. The time since cerebral infarction was 18.6 ± 6.7 and 17.4 ± 6.2 days in the IPA + TOT and TOT groups, respectively. None of the patients’ characteristics were different between these two groups (all *p* > 0.05, Table 1).

**Table 1:** Clinical characteristics of IPA + TOT and TOT groups.

Clinical characteristics	IPA + TOT group (*N* = 105)	TOT group (*N* = 103)	*p* value
Age (years), mean ± SD	64.6 ± 8.7	63.0 ± 9.3	0.227
Male, no. (%)	70 (66.7)	66 (64.1)	0.695
Education, no. (%)			0.654
Primary school	49 (46.7)	52 (50.5)	
Junior and senior high schools	40 (38.1)	36 (35.0)	
University and above	16 (15.2)	15 (14.5)	
BMI (kg/m^2^), mean ± SD	24.7 ± 3.5	24.9 ± 3.1	0.688
History of diseases, no. (%)			
Hypertension	82 (78.1)	74 (71.8)	0.298
Hyperlipidemia	53 (50.5)	46 (44.7)	0.401
Diabetes	26 (24.8)	26 (25.2)	0.936
Time since cerebral infarction (days), mean ± SD	18.6 ± 6.7	17.4 ± 6.2	0.209
Hemiplegic sites, no. (%)			0.406
Left	56 (53.3)	49 (47.6)	
Right	49 (46.7)	54 (52.4)	

IPA, infrared irradiation in predefined acupoints; TOT, task oriented training; BMI, body mass index; SD, standard deviation.

### 3.2 Comparison of neurological function and quality of life between groups at week 3

The NIHSS score was used for the assessment of neurological function. At baseline, the NIHSS score was not different between the IPA + TOT and TOT groups (*p* = 0.410). At W3, the NIHSS score was not significantly different between these two groups; however, the NIHSS score was seemingly lower in the IPA + TOT group than in the TOT group (*p* = 0.268, Table 2). The SS-QOL scale was used to assess quality of life. At baseline, the SS-QOL scale score was similar between these two groups, whereas at W3, the SS-QOL score was higher in the IPA + TOT group than in the TOT group (*p* = 0.006, Table 2). Besides, the change value of NIHSS score and SS-QOL from baseline to W3 was calculated using their values at W3 minus that at basline, and these change values were compared between the IPA + TOT and TOT groups. It showed that the change values of NIHSS score were similar between groups, while the change value of SS-QOL was greater in the IPA + TOT compared with TOT group (*p* < 0.001, Table 2). After FDR adjustment, the significance did not change (Supplementary Table 1). Furthermore, the changes of NIHSS score and SS-QOL from baseline and W3 within each group were also reported. It was shown that the NIHSS score and SS-QOL showed a significant improvement from baseline to W3 in each group (all *p* < 0.001, Table 2).

**Table 2:** Clinical outcomes.

Measure	IPA + TOT group (*N* = 105)	TOT group (*N* = 103)	*p*^1^ value
NIHSS score			
Baseline, mean ± SD	15.6 ± 4.5	15.3 ± 4.7	0.410
W3, mean ± SD	8.0 ± 2.5	8.6 ± 3.1	0.268
Change value, mean ± SD	−7.6 ± 4.7	−6.7 ± 4.2	0.212
*p*^2^ value	<0.001	<0.001	
Barthel index			
Baseline, mean ± SD	32.7 ± 11.5	33.9 ± 12.7	0.426
W3, mean ± SD	59.2 ± 17.6	51.7 ± 16.5	0.011
Change value, mean ± SD	26.4 ± 18.6	17.8 ± 17.1	0.003
*p*^2^ value	<0.001	<0.001	
FMA for upper limb			
Baseline, mean ± SD	19.2 ± 10.1	20.1 ± 8.7	0.396
W3, mean ± SD	32.2 ± 12.4	28.2 ± 13.1	0.028
Change value, mean ± SD	12.7 ± 15.4	8.1 ± 14.2	0.026
*p*^2^ value	<0.001	<0.001	
FMA for lower limb			
Baseline, mean ± SD	10.1 ± 4.6	10.3 ± 4.2	0.562
W3, mean ± SD	18.0 ± 4.1	16.1 ± 4.7	0.002
Change value, mean ± SD	7.9 ± 5.8	5.8 ± 4.6	0.005
*p*^2^ value	<0.001	<0.001	
SS-QOL			
Baseline, mean ± SD	108.3 ± 27.3	109.4 ± 26.0	0.774
W3, mean ± SD	142.1 ± 40.2	128.8 ± 35.0	0.006
Change value, mean ± SD	33.7 ± 26.5	19.4 ± 16.4	<0.001
*p*^2^ value	<0.001	<0.001	

IPA, infrared irradiation in predefined acupoints; TOT, task oriented training; BMI, body mass index; SD, standard deviation; W3, week 3. NIHSS, national institutes of health stroke scale; FMA, Fugl–Meyer assessment; SS-QOL, stroke-specific quality of Life. *p*^1^, between-group comparison. *p*^2^, within-group comparison between baseline and week 3.

### 3.3 Comparison of activities of daily living and limb motor function between groups at W3

**Figure 1:**
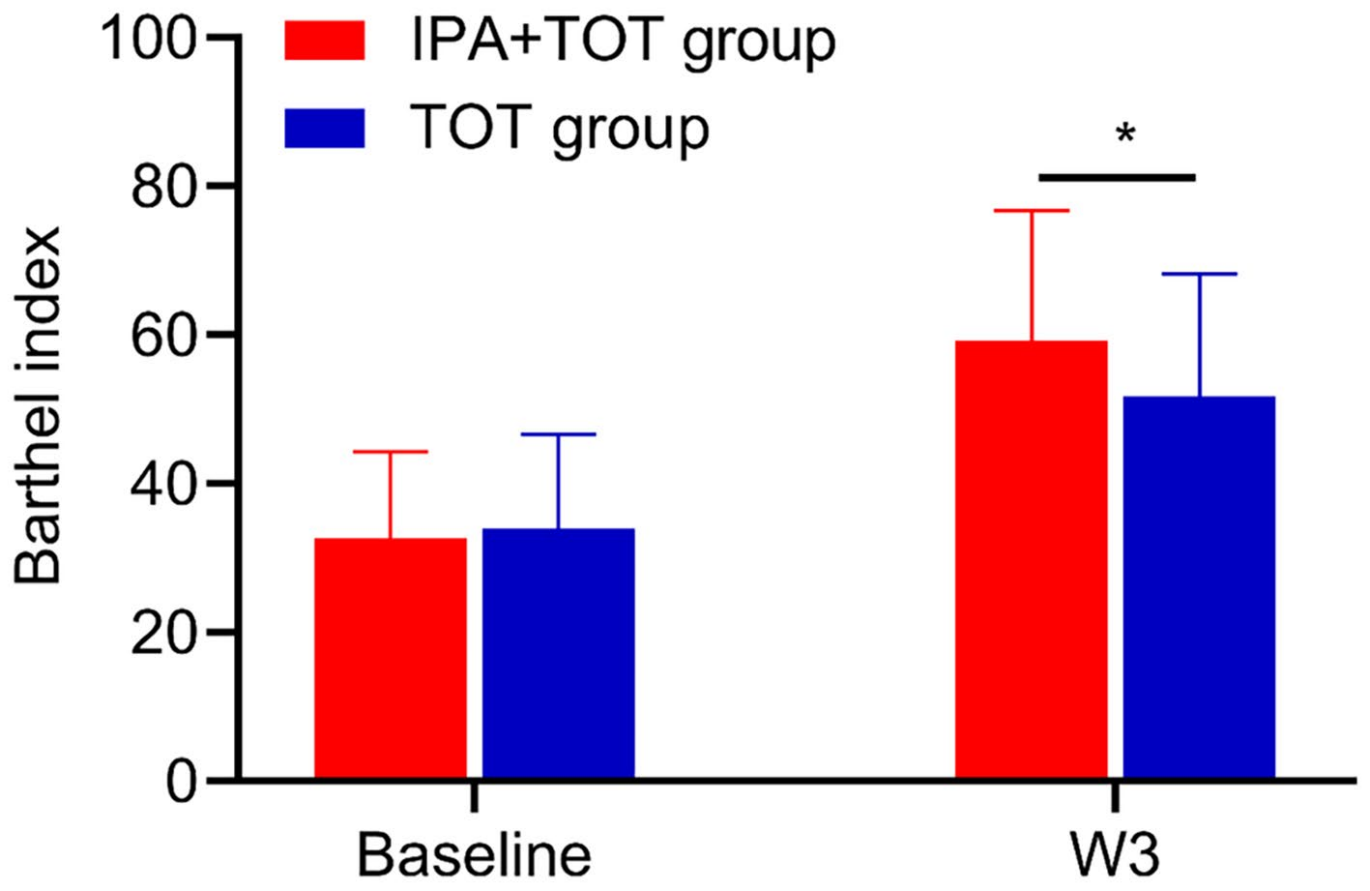
Comparison of the Barthel index between the IPA + TOT group and the TOT group at baseline and W3. **p < 0.01.

The barthel index was used to assess activities of daily living. The barthel index was similar between these two groups (*p* = 0.426), while it was greater in the IPA + TOT group than in the TOT group at W3 (*p* = 0.011, Figure 1).

**Figure 2:**
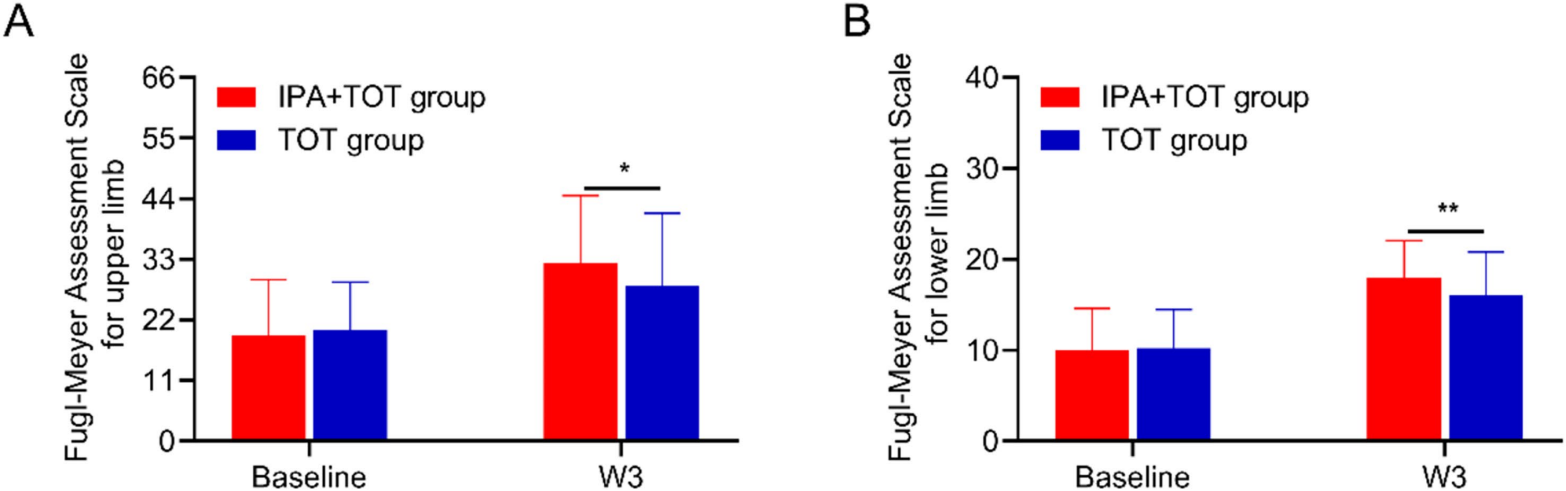
Comparison of Fugl–Meyer assessment scores between the IPA + TOT group and TOT group at baseline and W3. Comparison of Fugl–Meyer assessment scores for the upper limb **(A)** and lower limb **(B)** between the IPA + TOT group and the TOT group at baseline and W3. *p < 0.05, **p < 0.01.

The FMA scale was used for the assessment of limb motor function. For the upper limb, the FMA score was not different between the two groups at baseline (*p* = 0.396), but it was greater in the IPA + TOT group than in the TOT group at W3 (*p* = 0.028, Figure 2A). Similarly, the FMA scale score for the lower limb was also greater in the IPA + TOT group than in the TOT group at W3 (*p* = 0.002, Figure 2B). Besides, the change value of Barthel index, FMA for upper limb, and FMA for lower limb from baseline to W3 was calculated using their values at W3 minus that at basline, and these change values were compared between the IPA + TOT and TOT groups. It showed that the change values of these three indices were greater in the IPA + TOT compared with TOT group (all *p* < 0.05, Table 2). After FDR adjustment, the significance did not change (Supplementary Table 1). Furthermore, the changes of Barthel index, FMA for upper limb, and FMA for lower limb from baseline and W3 within each group were also reported. It was shown that the all these three indices showed a significant improvement from baseline to W3 in each group (all *p* < 0.001, Table 2). Furthermore, the effect sizes for changes of these clinical outcomes were shown in Supplementary Table 2. These finding indicated that effect size of NIHSS score was small; the effect size of Barthel index, FMA for upper limb, and FMA for lower limb was moderate, while the effect size was high in SS-QOL score (Supplementary Table 2).

### 3.4 Comparison of the surface electromyographic signals between groups at W3

**Figure 3:**
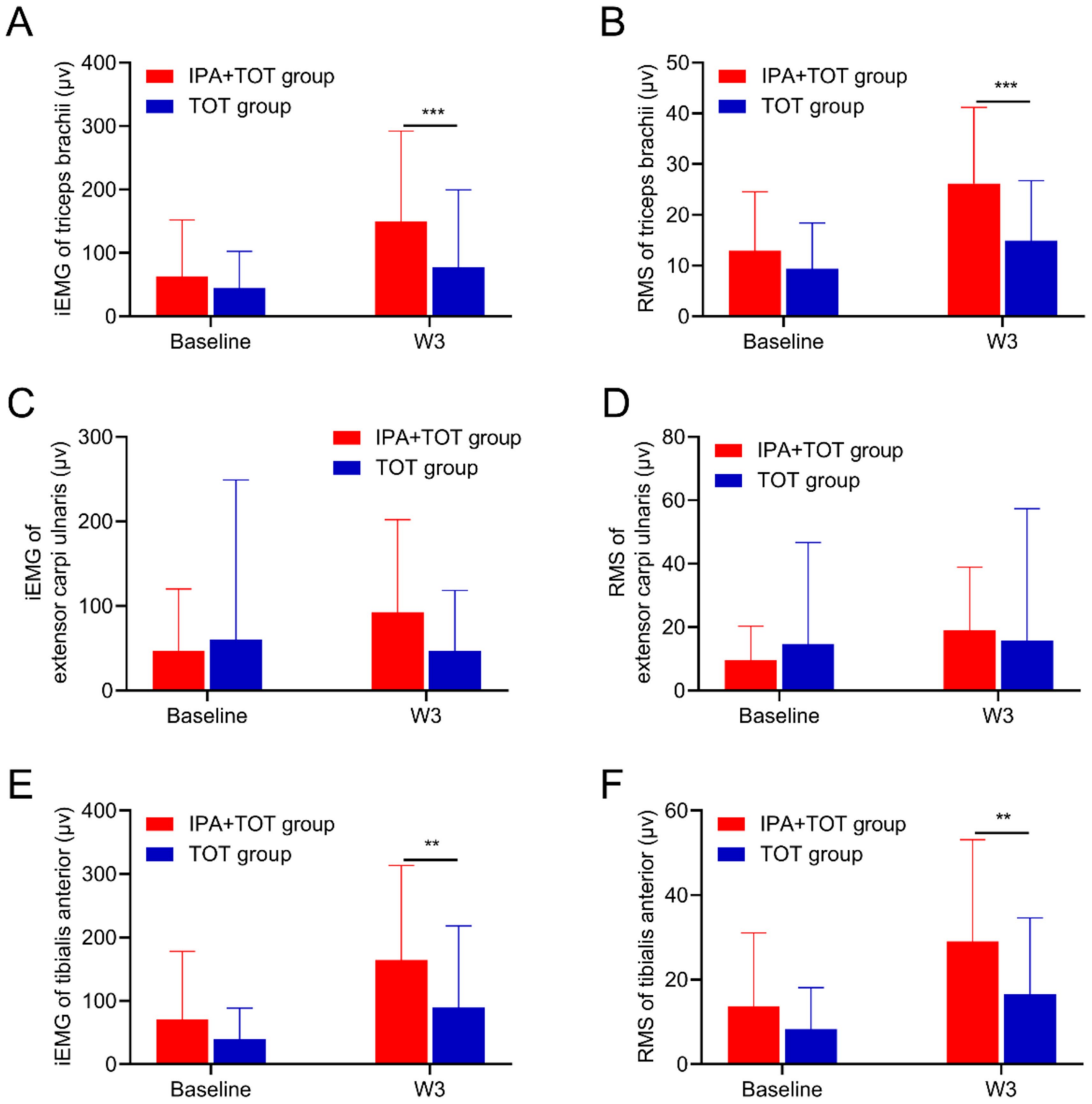
Comparison of electromyographic activity levels between the IPA + TOT group and TOT group at baseline and W3. Comparison of the electromyography integral value **(A)** and the root mean square value **(B)** for the triceps brachii between the IPA + TOT group and the TOT group at baseline and W3; comparison of the electromyography integral value **(C)** and the root mean square value **(D)** for the extensor carpi ulnaris between the IPA + TOT group and the TOT group at baseline and W3; comparison of the electromyography integral value **(E)** and the root mean square value **(F)** for the tibialis anterior between the IPA + TOT group and the TOT group at baseline and W3. *p < 0.05, **p < 0.01.

**Figure 4:**
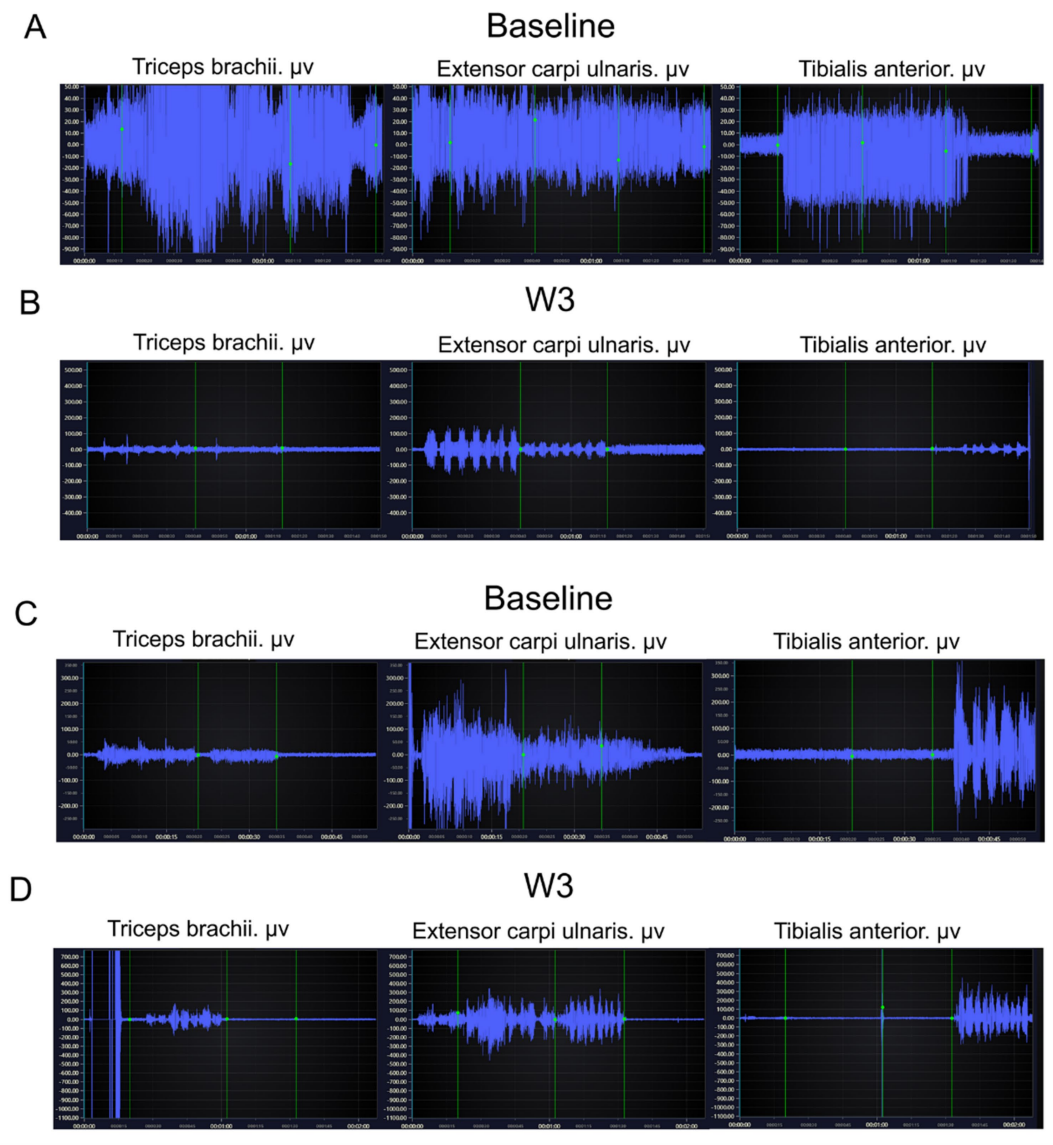
Illustration of surface electromyographic signals of the triceps brachii, extensor carpi ulnaris, and tibialis anterior in the IPA + TOT group and TOT group at baseline and W3. Images of surface electromyographic signals of the triceps brachii, extensor carpi ulnaris, and tibialis anterior at baseline **(A)** and W3 **(B)** in the TOT group. Images of surface electromyographic signals of the triceps brachii, extensor carpi ulnaris, and tibialis anterior at baseline **(C)** and W3 **(D)** in the IPA + TOT group.

The surface electromyographic signals in the triceps brachii, extensor carpi ulnaris, and tibialis anterior were detected and compared between the groups. The iEMG (*p* < 0.001, Figure 3A) and RMS (*p* < 0.001, Figure 3B) of the triceps brachii were greater in the IPA + TOT group than in the TOT group at W3. However, the iEMG (*p* = 0.107, Figure 3C) and RMS (*p* = 0.099, Figure 3D) of the Wrist extensor muscles were similar between the IPA + TOT group and the TOT group at W3. Furthermore, the iEMG (*p* = 0.002, Figure 3E) and RMS (*p* = 0.008, Figure 3F) of the tibialis anterior were both greater in the IPA + TOT group than in the TOT group at W3. The surface electromyographic signals are shown in Figures 4A–D. In detail, the baseline and W3 surface electromyographic signals for the TOT group are shown in Figures 4A,B, while the corresponding images for the IPA + TOT group are shown in Figures 4C,D. Besides, the change value of iEMG of Triceps brachii, RMS of Triceps brachii, iEMG of Wrist extensor muscles, RMS of Wrist extensor muscles, iEMG of tibialis anterior muscle, and RMS of tibialis anterior muscle from baseline to W3 was calculated using their values at W3 minus that at basline, and these change values were compared between the IPA + TOT and TOT groups. It showed that the change values of most of these surface electromyographic signals were greater in the IPA + TOT compared with TOT group (all *p* < 0.01), except that iEMG of Wrist extensor muscles was of no difference between groups (*p* = 0.050, Table 3). After FDR adjustment, the significance did not change (Supplementary Table 3). Furthermore, the changes of these indices from baseline and W3 within each group were also reported. It was shown that the all these indices showed a significant improvement from baseline to W3 in IPA + TOT and TOT groups (all *p* < 0.05, Table 3).

**Table 3:** Surface electromyographic outcomes.

Measure	IPA + TOT group (*N* = 105)	TOT group (*N* = 103)	*p*^1^ value
iEMG of triceps brachii			
Baseline, mean ± SD	63.2 ± 89.1	44.4 ± 58.2	0.149
W3, mean ± SD	149.2 ± 142.3	77.4 ± 122.1	<0.001
Change value, mean ± SD	86.0 ± 110.4	30.6 ± 113.7	<0.001
*p*^2^ value	<0.001	<0.001	
RMS of triceps brachii			
Baseline, mean ± SD	12.9 ± 11.6	9.4 ± 9.0	0.205
W3, mean ± SD	26.2 ± 15.0	14.9 ± 11.8	<0.001
Change value, mean ± SD	13.2 ± 10.2	5.1 ± 8.3	<0.001
*p*^2^ value	<0.001	<0.001	
iEMG of Wrist extensor muscles			
Baseline, mean ± SD	47.2 ± 72.9	60.4 ± 188.7	0.656
W3, mean ± SD	92.7 ± 109.4	47.1 ± 71.2	0.107
Change value, mean ± SD	45.5 ± 86.6	−14.7 ± 204.9	0.050
*p*^2^ value	<0.001	0.009	
RMS of Wrist extensor muscles			
Baseline, mean ± SD	9.6 ± 10.7	14.7 ± 32.0	0.325
W3, mean ± SD	19.1 ± 19.9	15.8 ± 41.6	0.099
Change value, mean ± SD	9.5 ± 12.8	0.6 ± 21.0	0.004
*p*^2^ value	<0.001	0.018	
iEMG of tibialis anterior muscle			
Baseline, mean ± SD	70.5 ± 107.8	39.9 ± 48.5	0.104
W3, mean ± SD	164.0 ± 149.5	89.6 ± 128.4	0.002
Change value, mean ± SD	93.6 ± 82.0	47.0 ± 99.0	0.001
*p*^2^ value	<0.001	<0.001	
RMS of tibialis anterior muscle			
Baseline, mean ± SD	13.7 ± 17.4	8.3 ± 9.9	0.205
W3, mean ± SD	29.1 ± 24.1	16.6 ± 18.1	0.008
Change value, mean ± SD	15.4 ± 13.2	7.8 ± 14.5	0.007
*p*^2^ value	<0.001	<0.001	

IPA, Infrared irradiation in predefined acupoints; TOT, task oriented training; BMI, body mass index; SD, standard deviation; W3, week 3. iEMG, integrated electromyography; RMS, root mean square; *p*^1^, between-group comparison. *p*^2^, within-group comparison between baseline and week.

Besides, the repeated-measures ANOVA was carried out to assess the main effects. It indicated that there was significant main effects of muscle for both iEMG (*p* < 0.001) and RMS (*p* = 0.005). The significant main effect of group was observed for RMS (*p* = 0.007), but not for iEMG (*p* = 0.070). Furthermore, there was no significant muscle × group interaction for iEMG (*p* = 0.409) or RMS (*p* = 0.935), indicating that treatment effects were consistent across the different muscles (Supplementary Table 4).

### 3.5 Correlation of clinical outcomes and EMG parameters

The association of clinical outcomes with the EMG parameters were determined. It indicated that Barthel index and SS-QOL were correlated with all EMG parameters (all *p* < 0.01, Supplementary Figure 1). The NIHSS score, FMA for upper limb, and FMA for lower limb were associated with iEMG and RMS for triceps brachii and Wrist extensor muscles (all *p* < 0.05), while they were not correlated with RMS for tibialis anterior muscle (*p* > 0.05, Supplementary Figure 1).

## 4 Discussion

Compared with TOT alone, IPA combined with TOT improved motor function, electromyographic activity level, daily living ability, and quality of life in cerebral infarction patients with hemiplegia. This result not only confirmed the clinical value of IPA + TOT as an auxiliary rehabilitation method but was also highly consistent with the research trends in recent years in terms of infrared irradiation, acupoint stimulation and TOT, which provided more evidence and methods for rehabilitation in cerebral infarction patients with hemiplegia.

In recent years, numerous studies have indicated that near/far-infrared irradiation can improve local microcirculation, enhance tissue oxygenation, promote nerve repair, and reduce muscle viscous resistance, thereby facilitating the recovery of limb function after stroke (16, 21, 22). The results of this study are highly consistent with these literature reports. Moreover, the advantages of irradiating fixed, custom acupoints are closely related to motor function and the electromyographic response of the triceps brachii and anterior tibial muscles. In addition, compared with research on improving motor function through invasive methods such as acupuncture and electroacupuncture at acupoints, this study revealed an increase in the electrical activity of key muscle groups in the limbs via noninvasive methods of the IPA, suggesting that neuroregulatory mechanisms related to acupoints also exist and play a role in infrared irradiation intervention (23–25). Consistent with the literature related to the TOT, this study confirmed that the TOT can promote motor learning and brain plasticity reconstruction (26, 27). However, when IPA was added as an auxiliary intervention, patients showed greater muscle excitability and motor participation when performing tasks, thereby further enhancing the training effect of TOT. This synergistic effect has been less reported in previous studies. This study provides new evidence to support this finding.

In terms of specific outcome indicators, the improvement in FMA scores with IPA combined with TOT was more prominent than that with TOT alone. Even though there was no definitive mechanism study reporting the potential mechanism, we hypothesized the potential explanation might be related to the enhanced tissue oxygenation, increased neural excitability, and improved peripheral feedback according to the previous studies (16, 21, 22). Previous experimental studies have suggested that activation of mitochondrial cytochrome C oxidase by infrared light may increase neuronal metabolic activity. This metabolic boost may underpin improved neural function and resilience, potentially facilitating more efficient task execution and learning during rehabilitation (28–30). The improvement in surface electromyographic signals observed in our study may be consistent with these proposed mechanisms; however, the present study did not directly assess the underlying biological pathways. Notably, the extensor carpi ulnaris did not significantly improve in this study, which was consistent with previous findings that the infrared light effect may be affected by the type of muscle fibers, the thickness of the subcutaneous soft tissue and the pattern of nerve innervation (22, 31), suggesting that the IPA has selective effects on different muscle groups.

This study also revealed that IPA combined with TOT could increase the Barthel index and SS-QOL score, indicating that the improvement in physical activity has been successfully translated into an increase in functional activity ability and further reflected in the quality of life. Compared with the findings in the literature that TOT has a limited impact on quality of life (32, 33), the greater improvement shown in this study may be related to the relief of muscle stiffness, reduction in fatigue and improvement in muscle control ability caused by infrared irradiation. These factors can all enhance patients’ ability to actively participate in rehabilitation training and daily activities, thereby increasing their subjective health perceptions.

Interestingly, although the motor function and SS-QOL reached statistical significance after the IPA + TOT treatment, the NIHSS score remained no difference between these two treatment modalities. We hypothesized the possible reasons might be that (1) the NIHSS score was a scale that evaluated multiple neural functions but not motor function, which mainly included consciousness, language, sensory function, and visual fields, and NHISS score was used mainly to evaluate the degree of nerve injury in the acute phase (34). (2) Moreover, the intervention period of this study was relatively short, making it difficult to determine the overall difference in the degree of nerve injury in the short term. (3) Besides, the intervention in this study mainly focused on the motor function improvement; therefore, only FMA and electromyography, but not NIHSS score, indicated a significant change in the current study. In addition, the effect sizes also showed the similar finding: benefit from the IPA + TOT was moderate to high regarding the quality of life and motor function compared with the TOT, while the benefit from IPA + TOT regarding the NIHSS score was low compared with the TOT. These findings suggested that IPA + TOT not only brought improvements regarding the motor function and quality of life statistically, but also contributed to the improvements with clinical significance.

Overall, the results of this study not only support the IPA as an effective auxiliary method for stroke rehabilitation but also suggest that the combined application of infrared irradiation and task-oriented training has a clear synergistic effect, which is of great clinical importance in promoting electromyographic activity, increasing exercise participation, and accelerating the recovery of motor function. As a safe, easy-to-operate and widely promotable physical factor therapy, IPA provides a new technical path for the combination of rehabilitation medicine and traditional Chinese medicine and offers a new feasible means for the systematic rehabilitation of stroke patients with hemiplegia. Compared with the existing adjunctive rehabilitation strategies, such as the acupuncture, functional electrical stimulation, transcranial magnetic stimulation, and photobiomodulation-based interventions, the IPA + TOT realized the similar efficacy outcomes, including the improvement on motor function, activities of daily living, and quality of life (35–37). However, due to that the noninvasive nature of IPA, it seems to be easier to apply in clinical practice and could reduce the unnecessary adverse reactions. However, there is still lack of the direct head to head comparison between the IPA + TOT with other adjunctive rehabilitation strategies. Further studies are still needed to verify this hypothesis.

This study also has certain limitations. First, this study lacked long-term follow-up, and it was still impossible to determine whether the therapeutic effect was sustainable or cumulative. Therefore, a study with longer follow-up period was needed to further verify the long-term benefit from IPA + TOT. Second, even though this study proposed the IPA at a certain setting, the optimal irradiation parameters of the IPA have not been clearly defined, including the power, irradiation time and acupoint combination, all of which need to be further optimized in future research. Third, this study was carried out in Shanghai, which mainly included Chinese patients, and the generalizability of the findings might be limited to all populations worldwide. Fourth, patients in the IPA + TOT group received an additional intervention that increased the overall treatment duration compared with TOT alone. Therefore, it could not be fully determined whether the observed benefits were attributable solely to the specific effect of IPA or were partially influenced by the increased treatment exposure. Fifth, there was no blind intervention between groups, which might induced the placebo effects and expectation bias.

In conclusion, IPA combined with TOT is associated with better motor function, electromyographic activity level, daily living ability, and quality of life in cerebral infarction patients with hemiplegia. However, further studies with longer follow-up periods and more rigorous study designs are required to confirm its long-term efficacy and clinical value.

## Data Availability

The original contributions presented in the study are included in the article/Supplementary material, further inquiries can be directed to the corresponding author.

## Supplementary material

Supplementary Figure 1 The association of clinical outcomes with the EMG parameters. **p* < 0.05, ***p* < 0.01, ****p* < 0.001, ns: non-significance.
