# Supplementary figures and images for: Efficacy of infrared irradiation at predefined acupoints combined with task-oriented training as a rehabilitation strategy in cerebral infarction patients with hemiplegia
Source: Front Neurol. 2026 Jul 17;17:1777129. doi: 10.3389/fneur.2026.1777129 (PMC13423720; doi:10.3389/fneur.2026.1777129)

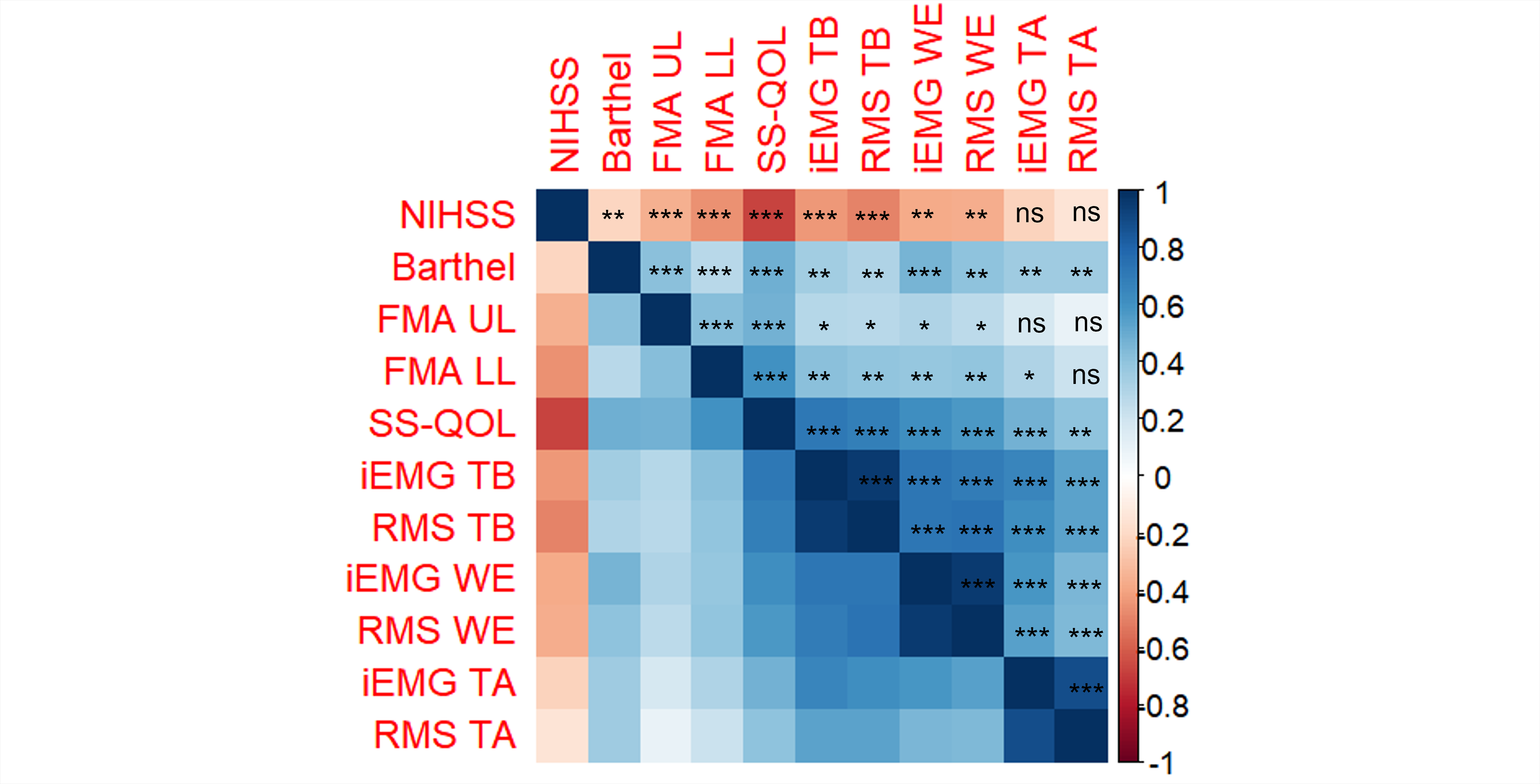

Supplement: Supplementary Figure 1 — The association of clinical outcomes with the EMG parameters. *p < 0.05, **p < 0.01, ***p < 0.001, ns: non-significance. [file Image_1.tif]
