# Supplementary material for: Efficacy of infrared irradiation at predefined acupoints combined with task-oriented training as a rehabilitation strategy in cerebral infarction patients with hemiplegia
Source: Front Neurol. 2026 Jul 17;17:1777129. doi: 10.3389/fneur.2026.1777129 (PMC13423720; doi:10.3389/fneur.2026.1777129)
Supplement: Supplementary file 2 [file Table_1.docx]

**Supplementary Table 1.** FDR correction of multiple comparisons for clinical outcomes.

|  | Raw *P* value | FDR-adjusted P value |
| --- | --- | --- |
| NIHSS | 0.212 | 0.212 |
| Barthel index | 0.003 | 0.008 |
| FMA for upper limb | 0.026 | 0.033 |
| FMA for lower limb | 0.005 | 0.008 |
| SS-QOL | <0.001 | 0.005 |

FDR, false discovery rate; NIHSS, National Institutes of Health Stroke Scale; FMA, Fugl-Meyer Assessment; SS-QOL, Stroke-Specific Quality of Life. P values were adjusted using the Benjamini–Hochberg FDR procedure.
