# Supplementary material for: Efficacy of infrared irradiation at predefined acupoints combined with task-oriented training as a rehabilitation strategy in cerebral infarction patients with hemiplegia
Source: Front Neurol. 2026 Jul 17;17:1777129. doi: 10.3389/fneur.2026.1777129 (PMC13423720; doi:10.3389/fneur.2026.1777129)
Supplement: Supplementary file 3 [file Table_2.docx]

**Supplementary Table 2.** Effect sizes for changes in clinical outcomes between groups.

|  | Mean difference of change values | 95% CI | Cohen's d |
| --- | --- | --- | --- |
| NIHSS | -0.9 | -2.17-0.27 | 0.20 |
| Barthel index | 8.6 | 3.78-13.54 | 0.48 |
| FMA upper limb | 4.6 | 0.75-8.85 | 0.31 |
| FMA lower limb | 2.1 | 0.64-3.50 | 0.40 |
| SS-QOL | 14.3 | 8.31-20.34 | 0.65 |

Effect sizes were calculated using Cohen’s d based on the change values from baseline to week 3. NIHSS, National Institutes of Health Stroke Scale; FMA, Fugl-Meyer Assessment; SS-QOL, Stroke-Specific Quality of Life.
