# Supplementary material for: Efficacy of infrared irradiation at predefined acupoints combined with task-oriented training as a rehabilitation strategy in cerebral infarction patients with hemiplegia
Source: Front Neurol. 2026 Jul 17;17:1777129. doi: 10.3389/fneur.2026.1777129 (PMC13423720; doi:10.3389/fneur.2026.1777129)
Supplement: Supplementary file 4 [file Table_3.docx]

**Supplementary Table 3.** FDR correction of multiple comparisons for Surface electromyographic outcomes.

|  | Raw *P* value | FDR-adjusted P value |
| --- | --- | --- |
| iEMG of Triceps brachii | <0.001 | 0.002 |
| RMS of Triceps brachii | <0.001 | 0.002 |
| iEMG of Wrist extensor muscles | 0.050 | 0.050 |
| RMS of Wrist extensor muscles | 0.004 | 0.006 |
| iEMG of tibialis anterior muscle | 0.001 | 0.002 |
| RMS of tibialis anterior muscle | 0.007 | 0.008 |

FDR, false discovery rate; iEMG, integrated electromyography; RMS, root mean square; P values were adjusted using the Benjamini–Hochberg FDR procedure.
