# Supplementary material for: Efficacy of infrared irradiation at predefined acupoints combined with task-oriented training as a rehabilitation strategy in cerebral infarction patients with hemiplegia
Source: Front Neurol. 2026 Jul 17;17:1777129. doi: 10.3389/fneur.2026.1777129 (PMC13423720; doi:10.3389/fneur.2026.1777129)
Supplement: Supplementary file 5 [file Table_4.docx]

**Supplementary Table 4.** Repeated-measures ANOVA of changes in electromyographic parameters of muscles.

|  | Effect | F | *P* value |
| --- | --- | --- | --- |
| iEMG |  |  |  |
|  | Muscle | 12.133 | <0.001 |
|  | Group | 3.406 | 0.070 |
|  | Muscle*group | 0.899 | 0.409 |
| RMS |  |  |  |
|  | Muscle | 5.601 | 0.005 |
|  | Group | 7.725 | 0.007 |
|  | Muscle*group | 0.067 | 0.935 |

iEMG, integrated electromyography; RMS, root mean square.
